# Identification and characterization of a high kernel weight mutant induced by gamma radiation in wheat (*Triticum aestivum* L.)

**DOI:** 10.1186/s12863-015-0285-x

**Published:** 2015-10-28

**Authors:** Xuejiao Cheng, Lingling Chai, Zhaoyan Chen, Lu Xu, Huijie Zhai, Aiju Zhao, Huiru Peng, Yingyin Yao, Mingshan You, Qixin Sun, Zhongfu Ni

**Affiliations:** State Key Laboratory for Agrobiotechnology and Key Laboratory of Crop Heterosis and Utilization (MOE) and Key Laboratory of Crop Genomics and Genetic Improvement (MOA), Beijing Key Laboratory of Crop Genetic Improvement, China Agricultural University, Yuanmingyuan Xi Road No. 2, Haidian District, Beijing, 100193 China; Department of Plant Genetics and Breeding, China Agricultural University, Beijing, 100193 China; National Plant Gene Research Centre, Beijing, 100193 China; Hebei Crop Genetic Breeding Laboratory Institute of Cereal and Oil Crops, Hebei Academy of Agriculture and Forestry Sciences, Shijiazhuang, 050035 China

**Keywords:** Wheat, Kernel weight, Mutant, SSR marker, QTL

## Abstract

**Background:**

Inducing mutations are considered to be an effective way to create novel genetic variations and hence novel agronomical traits in wheat. This study was conducted to assess the genetic differences between Shi4185 and its mutant line Fu4185, produced by gamma radiation with larger grain, and to identify quantitative trait loci (QTLs) for thousand kernel weight (TKW).

**Results:**

Phenotypic analysis revealed that the TKW of Fu4185 was much higher than that of Shi4185 under five different environments. At the genomic level, 110 of 2019 (5.4 %) simple sequence repeats (SSR) markers showed polymorphism between Shi4185 and Fu4185. Notably, 30 % (33 out of 110) polymorphic SSR markers were located on the D-genome, which was higher than the percentage of polymorphisms among natural allohexaploid wheat genotypes, indicating that mutations induced by gamma radiation could be a potential resource to enrich the genetic diversity of wheat D-genome. Moreover, one QTL, *QTkw.cau-5D*, located on chromosome 5DL, with Fu4185 contributing favorable alleles, was detected under different environments, especially under high temperature conditions.

**Conclusions:**

*QTkw.cau-5D* is an environmental stable QTL, which may be a desired target for genetic improvement of wheat kernel weight.

**Electronic supplementary material:**

The online version of this article (doi:10.1186/s12863-015-0285-x) contains supplementary material, which is available to authorized users.

## Background

Bread wheat (*Triticum aestivum* L.) is one of the most important food crops, which accounts for 20 % of the world’s calorie consumption and growers (http://faostat3.fao.org/home/E). With the ever-growing population in the world, a pressing objective is to increase wheat productivity, which could be dissected by improving wheat yield potential and raising the yield ceiling [1]. Wheat yield can be attributed to the integration of the number of fertile spikes per unit area, grains per spike, and the kernel weight. Among these three yield components, kernel weight is a highly heritable trait and has made significant contributions to yield potential in modern wheat breeding [2]. For example, TKW of Chinese wheat varieties has increased by 2.19 g every 10 years from 1940 to 2000 [3]. However, for the majority of the 20^th^ century, TKW was mainly improved by applying selection techniques over phenotypic measurements. These years, detection of QTLs with molecular markers has drawn more attention. Up to date, QTLs for TKW have been mapped on all 21 wheat chromosomes [4–9]. In addition, the grain size-controlling genes in rice showed a significant association with the orthologs in wheat for the TKW trait [10–12].

Increasing temperature is a major element of observed global climate change (https://www.ncdc.noaa.gov/indicators/). For wheat, global production is estimated to fall by 6 % for each °C of further temperature increase [13]. Thus, high temperature (heat) stress during grain filling becomes a major problem for almost all wheat production areas in temperate regions [14, 15] and developing heat-tolerant cultivars has become an important objective for breeders. Extensive research into the heat tolerance of wheat during grain filling revealed that kernel weight was more suited for screening than other traits evaluated [16–19]. Therefore, identification of DNA markers associated with kernel weight under post-anthesis high temperature stress would allow marker-assisted selection (MAS) and increase the efficiency for improving yield potential during breeding. Despite its importance, only a few QTL mapping studies of kernel weight have focused on heat stress.

A powerful approach for deciphering the biological functions of genes is to produce mutants with altered phenotypes or physiological responses. In wheat, chemical and ionizing radiation mutagenesis have been universally used to generate genetic variations for breeding researches and genetic studies. In total, 274 mutant varieties of wheat were developed by physical or chemical mutagens from 1930 to 2014 (http://www-naweb.iaea.org/nafa/pbg/). As a mutant induced by gamma radiation from Shi4185, Fu4185 showed higher kernel weight. This study aimed to analyze the genetic differences between Fu4185 and Shi4185 by SSR markers and to identify the genomic regions responsible for kernel weight in segregating populations derived from Shi4185 and Fu4185 under timely and late-sown conditions at different locations. This study will contribute to a better understanding of the genome-wide genetic variation and the stability of the QTL for kernel weight under heat stress.

## Methods

### Plant materials and field experiment

Shi4185 is an elite wheat cultivar in the Northern China winter wheat region. Fu4185 is a gamma radiation-induced mutant of advanced generations (M8), which was donated by Dr. Fengwu Zhao (Hebei Academy of Agricultural Sciences, China). F_2_ seeds were generated by self-pollinating of the F_1_ progeny of Shi4185 and Fu4185 in 2010. A total of 249 F_2_ individuals and the two parental lines were planted in the experimental station at Shangzhuang, Beijing (40°06′N, 116°11′E) in the autumn of 2011 (BJ-2011), and their derived F_2:3_ lines were grown at Gaoyi, Hebei (37°37′N, 114°35′E) (HB-2012) and Linfen, Shanxi (36°05′N, 111°30′E) (SX-2012) in the autumn of 2012 with three replicates. For spring sowing, the seeds of F_2_ populations were surface sterilized with 70 % ethanol for 30 s and 10 % NaClO for 10 min, and vernalized for 40 days in Petri dishes on wet filter paper at 4 °C in the dark after germination. After that, the seeds of two F_2_ populations were planted in BJ on February 17, 2014 (BJ-2014) and in SX on March 1, 2014 (SX-2014), respectively. The F_2_ seeds were space-planted (7.5 cm between plants), and each F_2:3_ line was hand-sown using a randomized complete block design in two-row plots of 1.5 m long row with 0.3 m spacing between the rows.

### Trait evaluation

Data of yield and yield contributing traits (plant height, spike length, spikelet number per spike, thousand kernel weight) of Shi4185 and Fu4185 that were planted at BJ in the autumn of 2011 were recorded using 30 plants (10 random plants from each replication) before harvest. After harvest, TKW of F_2_ individuals were determined. For the 249 F_2:3_ lines, bulked seeds of 10 random plants from the same line per replication were measured for TKW using a camera-assisted phenotyping system. This system was provided by Hangzhou Wanshen Detection Technology Co., Ltd. (Hangzhou, China).

### DNA extraction and SSR marker analysis

Genomic DNA was extracted from seedling young leaves using the protocol as described by Sharp et al. [20]. The DNA was precipitated again with isopropanol, washed twice with 70 % ethanol and dissolved in TE buffer. DNA quality was checked using 1 % agarose to make sure no noticeable degradation.

The parental lines were screened with a total of 2019 SSR markers (*gwm, wmc, barc, cfd, ksm, gdm and pk*). Primer sequences for most SSR markers are available at http://wheat.pw.usda.gov/GG2/index.shtml. Primer sequences for SSR markers designed in our lab are partially listed in Table 1. PCR amplifications were carried out in a 10 μL volume containing 40 ng genomic DNA, 1 μL 10× reaction buffer, 0.2 μL 10 mmol L^−1^ dNTPs, 1.0 μL primer, 1 U rTaq DNA polymerase (Takara, Dalian) and 5.7 μL ddH_2_O. The PCR was performed by initially denaturing the template DNA at 94 °C for 5 min, followed by 35 cycles at 94 °C for 45 s, 55 °C for 30 s, and 72 °C for 30 s, then terminated by a final extension for 10 min at 72 °C. PCR fragments were separated on 8 % non-denatured polyacrylamide gel electrophoresis (PAGE) and visualized by silver staining according to Marklund, Chaudhary et al. [21].

**Table 1:** Polymorphic SSR markers for genetic map construction

Markers	Forward primer (5′–3′)	Reverse primer (5′–3′)	Annealing temperature (°C)
*Xcau1022*	CCTAACCATCCAACCATAAGT	TTTCACGTACTCAAAAGTGG	55
*Xcau1053*	GGATGTACATTGAACAGTGCT	CCCACCCTACTCACTTCAA	55
*Xcau1074*	ACCTAAAATCTTCCCCCTACT	GCCAATTAATGCAGACTAGC	55
*Xcau1087*	GGAAAATCATGCACACATGG	CCCCACCCTACTCACTTCAA	58
*Xcau1118*	TGTAATCCGTCTCCTACCTTAT	GTCATACATGTCATCGGACTAC	55
*Xcau1132*	AGTCAATGAACAGAGCCATC	CTTGACCTAGATAGGGAAACAA	55

### Marker development and QTL analysis

International Wheat Genome Sequencing Consortium (IWGSC) (http://www.wheatgenome.org/) has published survey sequence assemblies of the 21 individual chromosomes of Chinese Spring (*Triticum aestivum* L.). Firstly, the SSR markers were developed by using the survey sequence assembly of chromosome 5DL. SSR markers were designed under the conditions that dinucleotide and trinucleotide repeats were more than 30 and 21 times, respectively, in the SSR-flanking regions. Secondly, *Aegilops tauschii* is the diploid progenitor of the D-genome of allohexaploid wheat, and its genome is an invaluable reference for wheat genomics [22]. Recently, the single nucleotide polymorphism (SNP) genetic map of *Aegilops tauschii*, was constructed, and the collinearity between *Brachypodium*, rice, and sorghum was established [23]. Therefore, comparative genomics approaches were employed to design SSR markers using the draft genome sequences, the extended SNP marker sequences and BAC scaffolds of *Aegilops tauschii* accession AL8/78. Information of polymorphic SSR markers for genetic map construction was listed in Table 1.

Linkage analysis of the marker was performed using JoinMap 4.0 software (http://www.kyazma.nl/index.php/mc.JoinMap/sc.General). According to the linkage distance between markers, a linkage map was drawn using MapChart v2.2 software (http://www.biometris.nl). Single marker analysis and QTL analysis by Composite interval mapping (CIM) method were performed by WinQTL Cart 2.5 software package [24]. The logarithm of the odds (LOD) threshold scores were calculated using 1,000 permutations [25], [26]. A QTL was declared when the LOD score was greater than 2.0.

## Results

### Phenotypic analysis

**Fig. 1:**
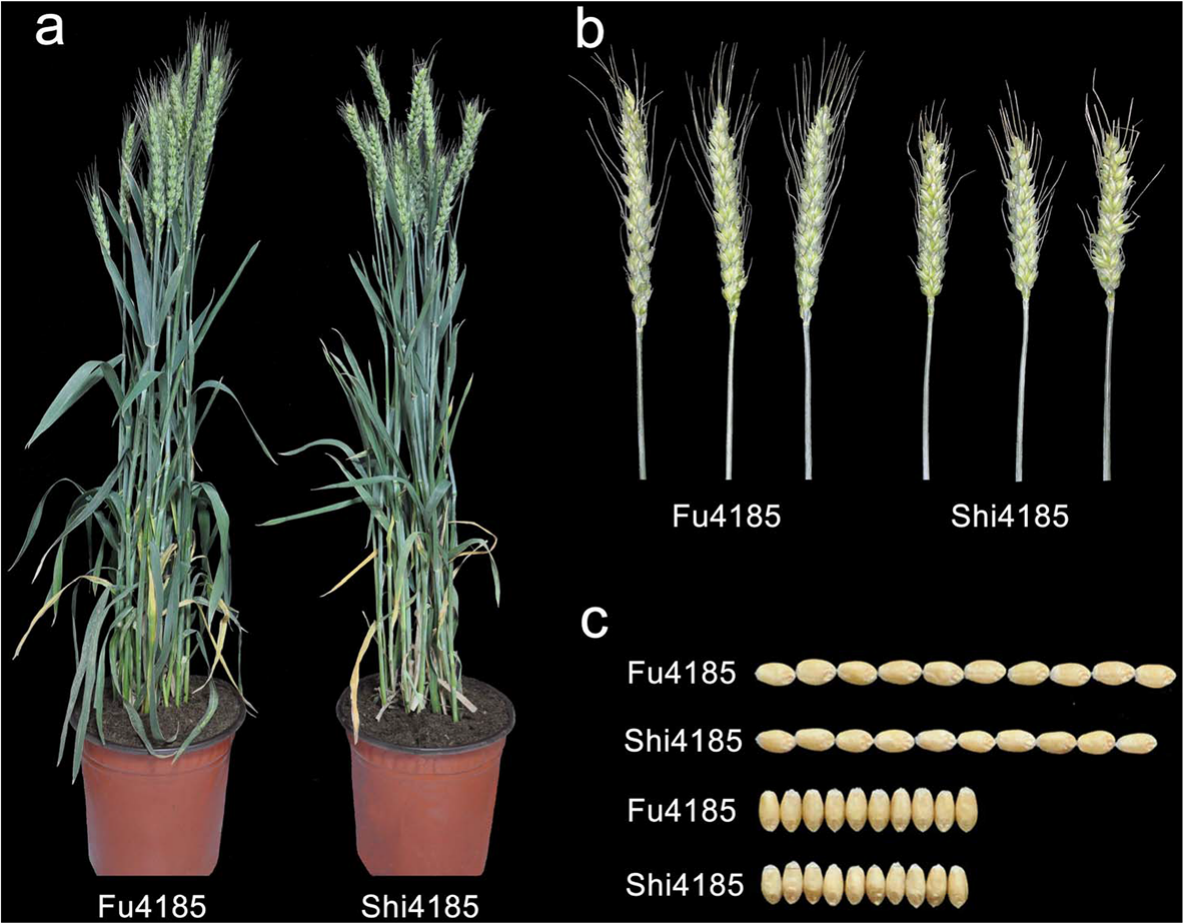
Phenotypic comparison of Shi4185 and Fu4185 planted at BJ-2011. **a** Plants at the filling stage; **b** Spikes at the filling stage; **c** Kernels after harvest

To characterize the phenotypic difference between Shi4185 and Fu4185, these two genotypes were firstly planted in BJ in the autumn of 2011 for analysis. As shown in Fig. 1, Shi4185 and Fu4185 showed significant differences in spike length and kernel size, whereas no difference was detected for plant height and spikelet number per spike. As expected, the TKW of Fu4185 was higher than that of Shi4185, which exhibited a good reproducibility in different environments (Table 2). In addition, there was a wide range of variations for both F_2_ and F_2:3_ populations, with coefficients of variation (CVs) ranging from 9.59 to 11.86 % in the F_2_ populations and from 4.0 to 4.87 % in the F_2:3_ populations, respectively. All populations showed continuous distributions with transgressive segregation on both sides for TKW (Additional file 1). Notably, the calculated broad-sense heritability (*h*_*B*_^2^) of TKW based on the data of F_2:3_ populations in HB-2012 and SX-2012 were 88.5 % and 86.9 %, respectively, suggesting that TKW is suitable for QTL mapping (Table 3).

**Table 2:** TKW (g) of Shi4185 and Fu4185 in different environments

Environment	Sowing season	Shi4185	Fu4185
BJ-2011	Autumn	36.01 ± 0.86	42.82 ± 0.75**
HB-2012	Autumn	33.51 ± 1.29	37.14 ± 1.10*
SX-2012	Autumn	32.23 ± 1.26	39.44 ± 0.70**
BJ-2014	Spring	33.76 ± 1.09	38.79 ± 1.20**
SX-2014	Spring	29.90 ± 1.10	36.61 ± 1.41**

*Indicates a significance level of *P* ≤ 0.05; **Indicates a significance level of *P* ≤ 0.01. All data are given as mean ± SD

**Table 3:** TKW (g) of F_2_ and F_2:3_ populations in different environments

Environment	Population	Range	Mean	SD	*h*_*B*_^2^ (%)
BJ-2011	F_2_	25.80–54.43	40.83	4.84	-
HB-2012	F_2:3_	32.85–40.59	36.24	1.45	86.9
SX-2012	F_2:3_	31.56–39.85	35.83	1.75	88.4
BJ-2014	F_2_	20.55–47.07	37.56	3.67	-
SX-2014	F_2_	23.64–44.05	34.60	3.32	-

**-**Indicates no data

### Genetic variations between Shi4185 and Fu4185

To investigate the genetic variations between Shi4185 and Fu4185, a total of 2019 SSR markers were used for analysis and 110 (5.4 %) markers were found to be polymorphic between Shi4185 and Fu4185. Two types of polymorphism were observed: presence or absence of the fragment (Type 1), and length differences of the fragment (Type 2). The numbers of the markers belong to Type 1 and 2 were 34 (30.9 %) and 76 (69.1 %), respectively. In addition, among the 110 polymorphic markers, 105 were located on 20 wheat chromosomes, with the exception of 4B. The numbers of polymorphic markers on each chromosome ranged from 1 (1A) to 14 (2B).

### Single-marker analysis

To identify molecular markers associated with TKW, a total of 249 F_2_ individuals, planted at BJ in the autumn of 2011, were genotyped using 58 polymorphic SSR markers. After the genotypic data of the F_2_ population was merged with the phenotypic data of the F_2_ and F_2:3_ populations, respectively, single-marker analyses were conducted using WinQTLCart version 2.5. The results showed that marker *Xbarc239* was significantly associated with TKW in both the F_2_ and the F_2:3_ populations at two different locations, whereas marker *Xgwm68* was only significantly linked with TKW in the F_2:3_ populations (Table 4).

**Table 4:** Single marker analysis of TKW in F_2_ and F_2:3_ populations

Environment	Population	marker	b0	b1	−2ln (L0/L1)	F (1, *n-2*)	Pr (F)
BJ-2011	F_2_	*Xbarc239*	40.94	−2.0	21.27	22.03	0.000 ****
HB-2012	F_2:3_	*Xbarc239*	36.28	−0.68	27.55	28.90	0.000****
		*Xgwm68*	36.26	−0.74	30.89	32.63	0.000****
SX-2012	F_2:3_	*Xbarc239*	35.87	−0.73	21.68	22.47	0.000****
		*Xgwm68*	35.84	−0.55	11.33	11.49	0.001***

***Indicates a significance level of *P* ≤ 0.001; ****Indicates a significance level of *P* ≤ 0.0001

**Fig. 2:**
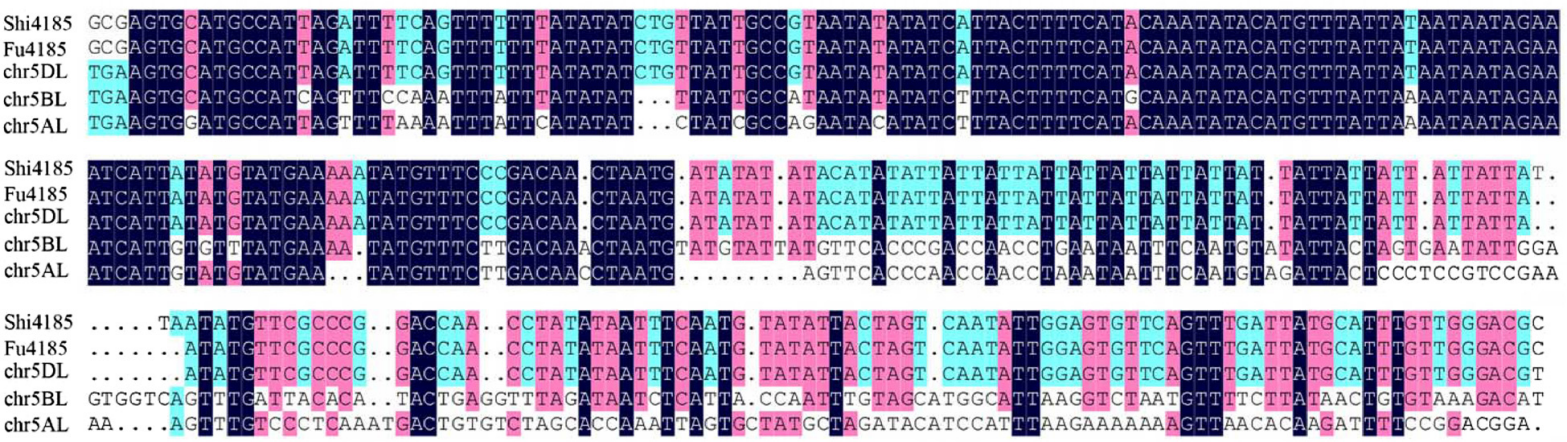
Sequence alignment of *Xbarc239* of Shi4185 and Fu4185 with wheat chromosome survey sequence

The genetic distance between *Xbarc239* and *Xgwm68* was 36.6 cM. Previous studies reported that *Xbarc239* was located on chromosome 5DL [27]. To verify the chromosome location of *Xbarc239*, the amplified products of the parental lines were cloned into the pEASY-blunt Simple Cloning Vector (Transgen) for sequencing and blasted against the wheat chromosome survey sequence database (http://www.wheatgenome.org/). The results showed that the amplified products exhibited much higher similarity (98.6 %) to chr5DL_ab_k71_contigs_4520158 as compared with chr5AL_ab_k95_contigs_l_2810952 and chr5BL_ab_k71_contigs_10879865 (56.1 % and 66.7 %, respectively), suggesting that *Xbarc239* was located on chromosome 5DL (Fig. 2, Additional file 2).

### Genetic linkage map construction of chromosome 5DL and QTL mapping

**Fig. 3:**
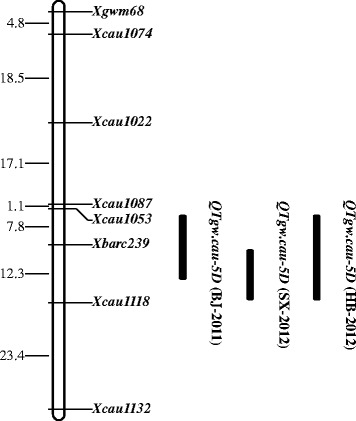
Locations of QTL for TKW in the F_2_ and F_2:3_ populations. QTLs with a LOD value more than 2.0 are shown on the right. The most likely intervals of the loci with LOD from top to 1.0 are shown in black

To further explore polymorphic markers within the QTL region of TKW on chromosome 5DL, 137 SSR markers were developed using the wheat survey assembly sequence and the draft genome sequences of *Aegilops tauschii*, among which 26 markers showed polymorphism between Shi4185 and Fu4185. These polymorphic markers were used for linkage map construction by genotyping 249 individuals of the F_2_ population (BJ-2011). Finally, one genetic linkage map of chromosome 5DL was constructed, including 8 SSR markers and spanning a total length of 84.96 cM (Fig. 3).

For QTL mapping, the genotype data of F_2_ population were merged with the phenotype data of both F_2_ and two F_2:3_ populations, respectively. Though composite interval mapping, one QTL for TKW (*QTkw.cau-5D*) was detected besides *Xbarc239*, with Fu4185 contributing favorable alleles (Fig. 3). For F_2_ population, the Fu4185 allele at *QTkw.cau-5D* increased TKW by 2.65 g, explaining 10.61 % of the phenotypic variation. For the two F_2:3_ populations, the Fu4185 allele at *QTkw.cau-5D* increased TKW by 0.45 to 0.56 g, explaining 1.58 to 2.94 % of the phenotypic variation (Table 5).

**Table 5:** Additive effects of QTL for TKW in F_2_ and F_2:3_ populations

Environment	Population	Marker interval^a^	Position (cM)^b^	LOD	Additive effect^c^	Contribution (%)
BJ-2011	F_2_	*Xcau1053*-*Xbarc239*	6.01	5.23	−2.65	10.61
HB-2012	F_2:3_	*Xcau1053*-*Xbarc239*	7.81	2.28	−0.45	2.94
SX-2012	F_2:3_	*Xbarc239*-*Xbarc1118*	5.98	2.27	−0.56	1.58
BJ-2014	F_2_	*Xcau1087-Xbarc239*	5.05	4.97	−1.70	9.51
SX-2014	F_2_	*Xcau1053-Xbarc239*	0.00	2.58	−1.43	14.87

^a^Marker interval is the interval containing the significant peak value of the QTL

^b^Position means the distance of the significant peak value for the QTL from the first marker in the marker interval

^c^A positive value indicates the Fu4185 allele having a positive effect on TKW, and negative value indicates Shi4185 allele having positive effect on TKW

To further assess the environmental stability of *QTkw.cau-5D*, another two F_2_ populations derived from Fu4185 and Shi4185 were grown at two locations during the spring season of 2014. Four SSR markers (*Xcau1053, Xcau1087, Xbarc239 and Xcau1118*) were used to genotype the two populations. Genetic linkage map construction and composite interval mapping were carried out for each F_2_ populations. QTL mapping analysis showed that *QTkw.cau-5D* could be detected in both the two populations, with Fu4185 contributing favorable alleles. The additive effects ranged from 1.43 to 1.70 g, explaining 9.51 to 14.87 % of the phenotypic variation (Table 5).

## Discussions

### Frequency, pattern and chromosome location of mutation at microsatellite loci between Shi4185 and Fu4185

Microsatellites or SSRs are composed of tandemly repeated, simple DNA sequence motifs of 1–6 nucleotide bases in length. Microsatellite markers are important tools for plant breeding, genetic and evolution studies [28]. A number of studies in animals and plants have calculated the natural mutation rate of microsatellites, and showed that the mutation rate varies greatly among species, ranging from 5 × 10^−6^ in Drosophila [29–31] to 10^−3^ in human [32, 33]. Gamma radiation can cause different types of mutations such as deletions, insertions, inversions and single-base substitution [34], but few studies have analyzed the frequency, polymorphism and pattern of microsatellite between the mutants and wild types in plants. In this study, SSR markers were employed to calculate the mutation frequencies induced by gamma radiation. Among the 2019 SSR markers, 110 (5.4 %) showed polymorphism between Shi4185 and Fu4185, which is lower than the percentage of polymorphism among natural allohexaploid wheat genotypes [35], but higher than the natural mutation rate of microsatellites per generation in different species [36]. In addition, two types of polymorphism were observed for SSR markers, including presence or absence of fragments (type 1) and fragment length differences for SSR markers (type 2). Multiple factors contribute to the observed variations. Type 1 might be produced by deletions, insertions, inversions and point mutations in the primer binding regions, whereas type 2 might be caused by insertion or deletion of bases in the SSR repeat regions. Consistent with this hypothesis, we sequenced marker *Xbarc239* and this mutation proved to be a change in the number of TTA repeats.

The knowledge of chromosome location for polymorphic SSR markers between Shi4185 and Fu4185 is critical for the mutation research, because it could enable us to relate the SSR loci with the altered phenotypes. Our data showed that 105 of 110 polymorphic SSR markers were distributed on 20 chromosomes (except 4B). The numbers of polymorphic markers on each chromosome ranged from 1 (1A) to 14 (2B), suggesting that mutation frequency are not uniform across the genome. Although gamma radiation induced a large number of mutations on the genome, only a few agronomical traits showed differences between Fu4185and Shi4185. Moreover, only 2 of 110 polymorphic SSR markers were significantly associated with TKW. Collectively, we speculated that most mutation events may not result in noticeable phenotypic changes, indicating that the wheat genome could largely bear the mutations.

Hexaploid common wheat (*Triticum aestivum* L.; genome AABBDD) evolved by natural hybridization of emmer wheat (*Triticum turgidum* L.; genome AABB) and *Aegilops tauschii* Coss. (genome DD) [37, 38]. Only a few *Aegilops tauschii*’s intraspecific lineages contributed to the evolution of common wheat, which resulted in relatively narrow genetic variation on the D-genome in wheat [39–42]. Consistently, the average genetic diversity of D-genome in bread wheat was lower than that of *Aegilops tauschii* [43]. Interestingly, we found that 33 of 110 polymorphic SSR markers were located on D-genome. Moreover, the QTL of kernel weight was mapped on chromosome 5DL, with Fu4185 contributing favorable alleles. Taken together, we proposed that mutants induced by gamma radiation could be a potential resource to enrich the genetic diversity of wheat D-genome.

### Environmental stability of QTL on chromosome 5DL controlling wheat kernel weight

Kernel weight is a complex quantitative and important agronomic trait. Mapping QTLs is the preliminary work for the genetic improvement by MAS [44]. To date, many studies have identified QTLs controlling kernel weight in common wheat cultivars and the QTLs were assigned to various chromosomes [4–9]. For example, Simmonds et al. [45] reported that the effect of yield QTL, located on chromosome 6A, was driven primarily by increased kernel weight due to wider grains, indicating that the enhancement of kernel weight by MAS may benefit the genetic improvement of wheat yield. In this study, QTL mapping for TKW was conducted using F_2_ and F_2:3_ populations derived from Shi4185 and Fu4185. One major QTL (*QTkw.cau-5D*), with Fu4185 contributing favorable alleles, was consistently detected on chromosome 5DL under different environments, which was linked to SSR marker *Xbarc239*. However, phenotypic variation explained by *QTkw.cau-5D* was not very stable under different environments, with a range of 1.58 to 14.87 %. Moreover, the effect of *QTkw.cau-5D* in F_2:3_ were smaller than in F_2_ populations. These results may be partially explained by the following reasons: 1) Effects of other QTLs that were not detected; 2) Inaccurate phenotypic data of each genotype in F_2_ populations for quantitative trait; 3) Impact of severe weather on TKW of F_2:3_ populations. According to the weather record, we speculated that excessive rainfall at the late stage of wheat grain filling in HB-2012 and SX-2012 may result in the smaller effect of *QTkw.cau-5D* in F_2:3_ as compared to F_2_ populations.

Heat stress is one of key abiotic stress affecting wheat production in temperate regions [14, 15]. In traditional breeding, membrane thermostability and chlorophyll fluorescence were used as indicators of heat-stress tolerance in wheat, as they showed strong genetic correlation with grain yield, but the method was a time and labor-consuming process [46]. Mapping QTLs linked to heat stress tolerance traits will help to develop wheat cultivars suitable for high-temperature environments through MAS strategy. Recently, different traits like grain filling duration (GFD), thousand kernel weight (TKW) and yield have proved to be the preferable criteria to screen for heat-tolerant wheat in fields and many QTLs with significant effects on heat tolerance have been detected [17, 18, 47]. QTLs can be categorized according to the stability of their effects across environmental conditions: a “constitutive” QTL is consistently detected across most environments; an “adaptive” QTL is detected only under specific environmental conditions [48, 49]. An important prerequisite for a successful MAS program aimed at improving heat tolerance is the identification of the “constitutive” QTLs. Recently, timely and late-sown were used for mapping QTLs associated with heat tolerance in wheat and two “constitutive” QTLs were detected on chromosome 2B and 7B [18]. Applying this method, we tested the effects of *QTkw.cau-5D* under different environments. Although the number of the days with the maximum temperature above 30 °C from flowering to harvesting at spring sowing season was much more than that at autumn sowing season (Table 6), *QTkw.cau-5D* was also detected under high temperature environments, which provide further evidence that it is an environmental stable QTL. However, to determine the accurate effect of *QTkw.cau-5D* under heat stress, it is better to compare spring and autumn planted trials in the same year, which deserved for further investigation.

**Table 6:** List of environment, flowering date, maturity date and No. of days for different daily maximum temperature ^a^ (Tmax) during grain filling

Environment	Flowering date	Maturity date	No. of days for different Tmax during grain filling			
			<30 °C	30–34 °C	>35 °C	Sum
BJ-2011	May 5, 2012	June 13, 2012	24	16	0	40
HB-2012	May 1, 2013	June 5, 2013	21	12	2	35
SX-2012	May 5, 2013	June 9, 2013	18	13	5	36
BJ-2014	May 11, 2014	June 20, 2014	16	21	4	41
SX-2014	May 15, 2014	June 25, 2014	9	29	4	42

^a^Data of Tmax from individual year of each environment (http://www.tianqi.com/)

**Fig. 4:**
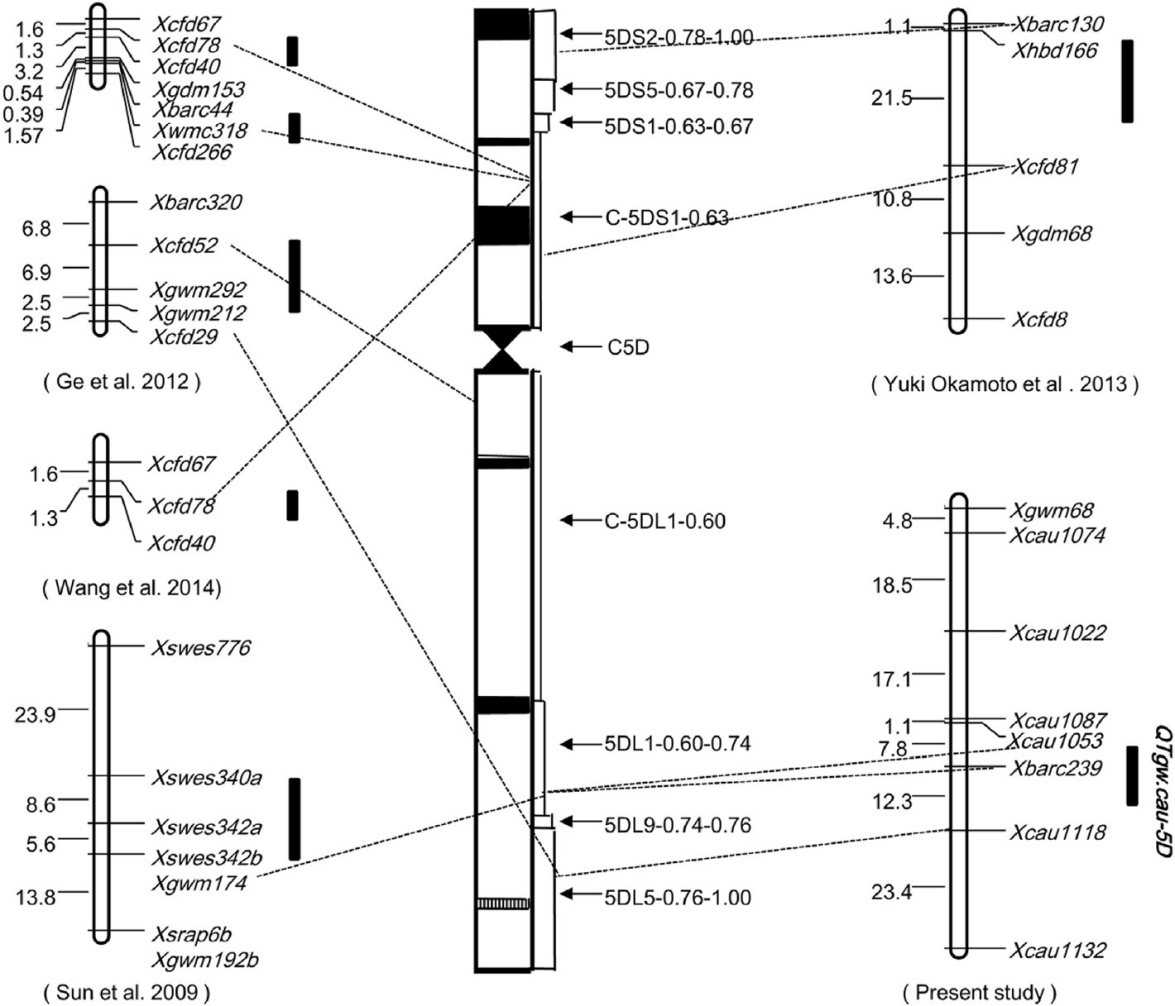
Physical map of QTLs for TKW. The most likely intervals or associated markers of the QTL are shown in black

In this study, the marker *Xbarc239* linked to *QTkw.cau-5D* was physically located to the bin of 5DL1-0.60-0.74 using Chinese Spring deletion lines [27]. When comparing previous results [50–53], we found that *QTkw.cau-5D* was located to the same bin as the QTL for TKW between *Xswes340a* and *Xswes342b* (*Xgwm174*) [51] (Fig. 4). Although further analysis was needed to clarify the relationship of these two QTLs, *QTkw.cau-5D* detected in our study may be a more desired target for genetic improvement of wheat kernel weight. Firstly, *QTkw.cau-5D* is an environmental stable QTL, whereas the QTL on chromosome 5D reported by Sun *et al.* [51] was only detected in 2 of 4 experimental environments; Secondly, the donor parent of Fu4185 (Shi4185) is an elite wheat cultivar in the Northern China winter wheat region. Moreover, a number of new cultivars derived from Shi4185 have been released in China, such as Henong130, Baomai10 and Nongda399. Theoretically, Fu4185 is a potential elite parent for genetic improvement of kernel weight in wheat breeding; Finally, considering the severe effect of heat stress on kernel weight in wheat production, our identified QTL associated with kernel weight under high temperature stress may increase the efficiency for improving wheat yield potential by MAS.

## Conclusions

One favorable and environmental stable QTL allele on chromosome 5DL controlling kernel weight was identified in Fu4185, a mutant of an elite wheat cultivar Shi4185 induced by gamma radiation. Furthermore, 30 % (33 of 110) polymorphic SSR markers between Shi4185 and Fu4185 were located on the D-genome. Taken together, these data revealed that mutations induced by gamma radiation could be a potential resource to enrich the genetic diversity of wheat D-genome.

## Additional files

Additional file 1:
**The data of genotype and phenotype.** (XLSX 100 kb) Additional file 2:
**The sequence of Xbarc239.** (TXT 1 kb)

## Abbreviations

QTL: Quantitative trait locus
TKW: Thousand kernel weight
SSR: Simple sequence repeats
MAS: Marker-assisted selection
PAGE: Polyacrylamide gel electrophoresis
IWGSC: International wheat genome sequencing consortium
SNP: Single nucleotide polymorphism
CIM: Composite interval mapping
LOD: Logarithm of the odds
CVs: Coefficients of variation
GFD: Grain filling duration
*h*_*B*_^2^: Broad-sense heritability
Tmax: Daily maximum temperature
